# The German version of the brief affective neuroscience personality scales including a LUST scale (BANPS–GL)

**DOI:** 10.3389/fnhum.2023.1213156

**Published:** 2023-07-07

**Authors:** Jürgen Fuchshuber, Theresa Prandstätter, Deborah Andres, Lisa Roithmeier, Beate Schmautz, Anton Freund, Andreas Schwerdtfeger, Human-Friedrich Unterrainer

**Affiliations:** ^1^Center for Integrative Addiction Research (CIAR), Grüner Kreis Society, Vienna, Austria; ^2^Department of Psychoanalysis and Psychotherapy, Medical University Vienna, Vienna, Austria; ^3^Institute of Psychology, University of Graz, Graz, Austria; ^4^Faculty of Psychology, University of Vienna, Vienna, Austria; ^5^Department of Psychiatry and Psychotherapeutic Medicine, Medical University Graz, Graz, Austria; ^6^Department of Religious Studies, University of Vienna, Vienna, Austria; ^7^Faculty of Psychotherapy Science, Sigmund Freud University, Vienna, Austria

**Keywords:** affective neuroscience, confirmative factor analysis, lust, primary emotions, scale adaptation

## Abstract

**Objectives:**

This study presents the German version of the Brief Affective Neuroscience Personality Scales (BANPS), which includes an additional subscale for the dimension LUST. The BANPS represents a shortened version of the Affective Neuroscience Personality Scales (ANPS), a self–report instrument to assess individual dispositions toward primary emotional systems as proposed by Jaak Panksepp.

**Methods:**

In a large sample (*N* = 926), the reliability and various facets of validity of the German translation of the BANPS were examined together with the newly included LUST scale. The BANPS–GL was related to the Big Five Inventory (BFI) and Sexual Sensation Seeking Scale (SSSS) and analyzed via confirmatory factor analysis (CFA).

**Results:**

Overall, the BANPS–GL exhibited reliabilities ranging from McDonald’s ω = 0.70 (CARE) to α = 0.86 (SADNESS) and plausible correlations with external criteria. For CFA a correlated 7–factor model demonstrated good fit [TLI = 0.95; RMSEA = 0.04 (90% CI: 0.04, 0.05); SRMR = 0.06]. A similar fit was demonstrated for a 2–higher–factor model [TLI = 0.93; RMSEA = 0.05 (90% CI: 0.05, 0.06); SRMR = 0.07].

**Conclusion:**

In broad agreement with the results of the original English version, the BANPS–GL showed good reliability and acceptable factorial validity, and overall improved the psychometric properties of the original long form. Finally, the inclusion of the dimension LUST allows for a complete coverage of the primary emotion dispositions as originally conceptualized by Panksepp.

## Introduction

Mammals are obviously more related in their emotional and motivational origins than one might initially think. Therefore, the neural pathways and corresponding neurochemistry that form the basis of our human emotional systems and trigger primary emotions are closely related in all mammals. These primary emotional action systems are evolutionarily and genetically anchored and provide mammals with a mechanism for survival (Panksepp, 1998; Panksepp and Biven, 2012). Furthermore, these systems are not static, but dynamic and capable of learning and adapting to new environmental influences and experiences throughout life (Davis and Montag, 2018).

The model of primary emotion systems was decisively developed within the framework of Affective Neurosciences (Panksepp, 1992). Based on cross–species research involving direct manipulation of neuronal structures, Panksepp (1998) postulated seven evolutionary basic emotion systems, namely SEEK(ING)/expectation, RAGE/anger, FEAR/anxiety, CARE/nurturance, PANIC/GRIEF/sadness, PLAY/joy and LUST/sexual pleasure, which can be assigned to specific neurophysiological networks (Montag et al., 2021). These systems are assumed to build the foundations of human personality development (Panksepp, 1998). Hence, Davis et al. (2003) developed the Affective Neuroscience Personality Scales (ANPS) which enables the psychometric assessment of individual primary emotion dispositions.

On a neurological level, all primary emotion systems extend from the midbrain via hypothalamus, medial thalamus and limbic system to various medial frontal cortex and ventral forebrain regions. In the midbrain, the periaqueductal gray (PAG) plays a central role in the archeological structure of our emotional life. The orbitofrontal cortex, located in the ventral forebrain regions, represents, among other things, a higher level of control over emotional reactivity (Panksepp and Biven, 2012; see Panksepp, 2011a for a concise review of neuronal substrates regarding specific primary emotion networks). All emotional affects are evaluative. They have a valence that is either *positive* and thus pleasant or *negative* and thus aversive. Therefore, emotions signal whether one should adopt an approaching or an avoiding attitude (Davis and Montag, 2018).

Behaviorally, the individual primary emotion systems are conceptualized as the following:

The SEEKING-network serves as the fundamental motivation system of mammal brains and ensures that individuals seek, find, and acquire all resources necessary for survival. It is crucially involved in the search for food and partners. The system drives exploratory and approach behaviors and promotes goal–directed activities. It controls reward learning through positive arousal, activating processes to achieve goals (Alcaro et al., 2007; Alcaro and Panksepp, 2011; Panksepp and Biven, 2012).

The primary emotion CARE is involved in caring for and raising offspring (Davis and Montag, 2018). It is considered a nurturing attitude, giving special attention to the care of people and animals in need. It exudes empathy and can be characterized by mental and physical affection (Panksepp, 1998; Davis et al., 2003).

A further positive emotion is PLAY, which is considered crucial for social bonding and the development of social skills. High expression in PLAY is indicated by humor, a playful nature, and strong social formations. It can be assumed that this trait is involved in the regulation of emotions in the neocortical areas (Davis et al., 2003; Siviy and Panksepp, 2011; Montag et al., 2021).

Furthermore, LUST is defined as a system representing male and female sexuality and the erotic sensations, sexual urges and pleasure that accompany it (Panksepp, 2003b, 2011b). Although LUST also represents a primary emotion, it was excluded from operationalization in the Affective Neuroscience Personality Scales (Davis et al., 2003), because the authors decided that it seemed less relevant to current conceptualizations of personality structure. In addition, it was assumed to be a possible affective component about which people do not want to show openness and honesty. Through the expectation of socially highly desirable reaction patterns there was the assumption, that it could influence and bias responses in the other scales (Davis et al., 2003; Davis and Panksepp, 2011; Montag et al., 2016). However, initial results by Fuchshuber et al. (2022) were able to show rather promising results regarding psychometric properties and general participant acceptance regarding a newly developed scale aiming to operationalize LUST.

The ANGER-network mediates aggressive behaviors in response to frustrations, restraints to the organism’s freedom of action or threatening stimuli. Emotional expression can also, *inter alia*, be induced by the lack of expression of other primary emotion systems. For example, frustration of the SEEKING system may trigger ANGER. Likewise, high satisfaction of the SEEKING system leads to a reduction in ANGER (Panksepp and Biven, 2012). Loss of resources as well as social rejection, such as spurning of love and acceptance or repression at the hierarchical level, are also prone to trigger aggressive impulses (Panksepp and Biven, 2012).

The FEAR system warns us of physical harm and pain. When it detects immediate danger, it triggers a freeze or flight response. In general, manifestations of FEAR are worrying, difficulty in decision making and rumination (Blanchard et al., 2001; Davis et al., 2003; McNaughton and Corr, 2004).

The PANIC/GRIEF system, which is also referred to as SADNESS system, is related to the early experiences of infant separation–distress (Panksepp and Watt, 2011; Montag et al., 2021). Even though this mental pain system is connoted as a negative primary emotion, it promotes social bonding and solidarity through arousal in the absence of close caregivers and social contacts (Panksepp, 1998, 2003a; Damasio et al., 2000). Affects such as feelings of sorrow, the need to cry or depressive moods characterize this Network (Panksepp, 2003b,^2006^; Panksepp and Biven, 2012). In psychometric studies, SADNESS showed consistently high correlations with FEAR, which psychologically might be explained with their concatenation via neuroticism or general negative emotionality (Barrett et al., 2013; Fuchshuber et al., 2019; Montag et al., 2022).

Previous studies employing the ANPS indicate that the primary emotion systems show a specific pattern in relation to the Big Five personality model. The strongest correlations are found between PLAY and Extraversion, CARE and Agreeableness, SEEKING with Openness, and the three factors with negative connotations (ANGER, FEAR, and SADNESS) with Neuroticism (Panksepp, 1998; Davis and Panksepp, 2011; Marengo et al., 2021). Here it can be noted in relation to the overlaps that the formulations of the FEAR scale are also aimed at worries and fears, whereby these strongly occurring correlations can be plausibly explained. Due to some problematic aspects of the original version–such as poorly worded items and problems regarding structural validity – to our knowledge at least two short versions were developed: The ANPS–S (Pingault et al., 2012) and the BANPS (Barrett et al., 2013). In the BANPS, the length of the survey instrument was minimized, intercorrelations of the scales were reduced, while their reliability was maintained or even improved (as in the case of SADNESS). The designation of the individual primary emotions and thus subscales were here set to SEEK for SEEKING, ANGER for RAGE, and SADNESS for PANIC/GRIEF (Barrett et al., 2013). While both versions significantly improved upon major issues of the original ANPS, research demonstrated superior psychometric properties of the BANPS, in particular regarding structural validity (Pedersen et al., 2014).

### Study aims

This study aims to translate and psychometrically evaluate a standardized measurement instrument covering all seven primary emotions. For this purpose, we examine the reliability and validity of a merger of BANPS (Barrett et al., 2013) and LUST scale (Fuchshuber et al., 2022). Examining a full primary affect model, we assume a similar model fit as Barrett et al. (2013). In this context, the new conceptualization provides a fundament for further research regarding the respective expressions and ramifications of human primary emotions systems.

## Materials and methods

### Sample and procedure

The investigated sample from the general population consisted of 926 German–speaking participants (gender: 72.7% female; age: 18–73 years, *M* = 28 years, SD = 9 years). According to the literature, since no possible exclusion criteria had been given so far, narrowing down the age and language was the only reason for exclusion, this results in in the criteria for participating in an age over 18 and speaking German at least fluently. From the original data set of 1,566 respondents, those who did not complete the questionnaire were excluded. Likewise, two persons who did not sign the consent form were subsequently deleted. Since virtual–based data collection is identical to paper–pencil testing in potential biases, reliability, and robustness (Gosling et al., 2004; Beuckelaer and Lievens, 2009), and both the BANPS (Barrett et al., 2013) and LUST scale (Fuchshuber et al., 2022) were administered online, this procedure was also implemented in this study. Participants were recruited through a student’s mail distribution list, advertisements in public forums and social networks.

The data was collected via the online–survey platform LimeSurvey©. Informed consent was obtained from all subjects before answering the questions. The survey consisted of various demographic questions (e.g., gender, age, education, and psychiatric diagnoses) as well as the standardized test procedures described below. The participants did not receive any personal compensation. However, there was a raffle of vouchers among all participants. Participants remained completely anonymous during and after the period of study participation. The study was carried out in accordance with the declaration of Helsinki. Ethical approval was granted by the ethics committee of the University of Graz, Austria.

### Translation process

For the use and translation of the BANPS, we obtained kind permission from the first author of the initial English version of the BANPS, Barrett et al. (2013). The sets of items corresponding to each facet of every BANPS domain were translated into German. Then, a bilingual person who had no prior knowledge of the instrument was given the translated items to create a back translation into English. After the back translation into English was created, copies of it were provided to the authors. The authors reviewed the back translation and proposed revisions, if necessary. This process was repeated until it was determined that the German version back translated into English was comparable to the original English instrument. The procedure with forward and backward translation as well as correspondence between linguistic as well as scientific experts, was applied according to Fenn et al. (2020).

### Psychometric assessment

After providing informed consent, participants received a demographic questionnaire asking for all personal data relevant to the study. The data sheet contained questions on gender, age, marital status, level of education and field of study, current occupation or training and sexual orientation as well as country of origin and language skills.

The *Brief–Affective Neuroscience Personality Scales* (BANPS; Barrett et al., 2013) represents the short form of the Affective Neuroscience Personality Scales (ANPS; Davis et al., 2003). While the ANPS has 112 items, the short form of it consists of 33 items. Responses are given on a five–point scale ranging from (1) strongly disagree to (5) strongly agree. The questionnaire is reliable and has good internal consistencies (0.74–0.86; Barrett et al., 2013). Test–retest reliabilities were measured at 6–week intervals and are also very high (0.82–0.94; Montag et al., 2021).

The *LUST-Scale* (Fuchshuber et al., 2022) was developed to measure an individual disposition toward the experience of sexual pleasure and eroticism (LUST). The 12–Item and the 5–Item versions show excellent to good internal reliability (L-12: Cronbach’s α = 0.90; L-5: α = 0.82). For this study the 5–item version of the L–Scale was used.

The *Big Five Inventory* (BFI; John et al., 1991) measures personality in terms of the five–factor model and thus Openness, Conscientiousness, Extraversion, Agreeableness and Neuroticism. The German version (Danner et al., 2016) of the inventory comprises 44 items on a 5–point Likert scale ranging from (1) strongly disagree to (5) agree very well. It achieves acceptable to good internal consistencies for young adults with Cronbach’s α between 0.71 and 0.85 across the five personality structures (Lang et al., 2001).

The *Sexual Sensation Seeking Scale* (SSSS; Hammelstein, 2005) found its origin in the *Sensation Seeking Scale* (SSS) designed by Zuckerman (1994). By reformulating the items of Sensation Seeking behaviors, such as thrill and adventure seeking or disinhibition, on a sexual level, they are characterized primarily by risk–taking behaviors. SSSS measures the propensity to seek new and varied sexual experiences and to take physical and social risks to achieve sexual satisfaction (Kalichman et al., 1994). The German version of the SSSS (Kalichman and Rompa, 1995) comprises eleven items on a four–point Likert scale, ranging answers from (1) not at all true for me up to (4) very true for me. The reliability of this scale is in the acceptable range with Cronbach’s α = 0.73.

### Statistical analyses

The statistical analysis was conducted via SPSS 29.0 and RStudio 2022.12.0 + 353. SPSS was used for data management and the calculations of descriptive statistics, reliabilities, exploratory factor analysis, MANOVA and bivariate correlations. The estimation of the confirmatory factor analysis was implemented with the R package Lavaan. A confirmatory factor analysis was performed which investigated a correlated 7–factor and a correlated 2–higher order factor solution. Furthermore, correlations, gender differences, descriptive statistics hierarchical multiple regressions as well as reliability of the BANPS–GL were assessed. For *post hoc*-tests of MANOVA Tukey-HSD was employed. Finally, we investigated scale invariance of the BANPS-GL comparing the factorial structure in female and male participants.

Sexual Sensation Seeking Scale (SSSS) and the Big Five Inventory (BFI) served as validation instruments for the new BANPS–GL. Regarding independent associations between BANPS-GL scales, BFI and SSSS hierarchical multiple regressions were performed, which investigated age and gender (step 1) and BFI and SSSS (step 2) as predictors of the BANPS-GL scales in order to assess independent associations between these constructs.

Due to the ordinally scaled items of the BANPS–GL CFA goodness–of–fit was assessed via diagonally weighted least squares (DWLS) estimation (Li, 2016). Following Kline (2015) the following cut–off values for acceptable global fit indices were applied: (1) Tucker–Lewis index relative fit index (TLI) >0.90; (2) the Square Root Error of Approximation (RMSEA) <0.08 and the upper bound of its 90% confidence interval <1; (3) Standardized Root Mean Square Residual (SRMR) <0.08. To control for α-inflation the level of significance was set to *p* < 0.01.

Measurement invariances was assessed as the following: In step 1 configural invariance of the model was tested for structural similarity structure similarity (i.e., the same number of factors, each represented by the same set of indicators). In the subsequent steps, model identification was achieved by constraining latent variables to unity. In Step 2, the equivalence of the factor loadings was tested by comparing the configural invariance model and the metric invariance model. Thereby the loadings between two groups are constrained to be equal while the other parameters are free to vary. In Step 3, the equivalence of intercepts was tested by comparing the metric invariance model with the intercept constrained scalar invariance model. In accordance with Cheung and Rensvold (2002) the criteria of −0.01 for ΔCFI was applied for invariance tests.

## Results

### Sample characteristics

The descriptive sample characteristics for the trial and validation phases are detailed in Table 1. The mean age of the participants was 28 (SD = 9 years). 648 (70%) of the participants were female, 21 (2%) stated “divers” as gender. Concerning sexuality, 696 (75.2%) were heterosexually oriented and with 121 (13.1%) the second most common indication corresponded to bisexuality. Regarding relationship status, most probands were single (*n* = 453; 48.9%), followed by the indication living in a relationship (*n* = 336, 36.3%). The majority had Austrian, German or Swiss nationality (*n* = 855, 92.3%). Most subjects’ highest educational qualification was a qualification for higher education (*n* = 415; 44.8%). Most of the persons are currently in education (*n* = 602, 65%), or in employment (*n* = 574, 62.9%). 130 subjects (14%) stated to be diagnosed with a psychiatric disorder. 209 (23.6%) take medication on a regular basis.

**TABLE 1:** Sample characteristics (exploration and validation phase).

Overall	*N* = 926
Gender	*N* = 648 Female (70%)
	*N* = 257 Male (28.8%)
	*N* = 21 Diverse (2.3%)
Age	*M* = 28 years (SD = 9 years)
Nationality	*N* = 855 AT, DE, CH, (92.3%)
	*N* = 51 EU (5.5%)
	*N* = 19 Non–EU (2.1%)
Relationship status	*N* = 453 Single (48.7%)
	*N* = 336 In relationship (36.3%)
	*N* = 110 Married (11.9%)
	*N* = 24 Divorced (2.6%)
	*N* = 3 Widowed (0.9%)
Sexual orientation	*N* = 696 Heterosexual (75.2%)
	*N* = 32 Homosexual (3.5%)
	*N* = 121 Bisexual (13.1%)
	*N* = 40 Pansexual (4.3%)
	*N* = 29 Asexual (3.1%)
	*N* = 8 Other (0.9%)
Education	*N* = 415 High-school diploma (44.8%)
	*N* = 214 Bachelor degree (23.1%)
	*N* = 143 Master degree (15.1%)
	*N* = 100 Apprenticeship (10.8%)
	*N* = 21 Doctoral degree (3.1%)
	*N* = 32 Compulsory school (3.5%)
	*N* = 1 No degree (0.1%)
Psychiatric disorders	*N* = 796 No (86%)
	*N* = 130 Yes (14%)

### Confirmatory factor analysis of the BANPS–GL

The correlated 7–factor model demonstrated an overall good fit with TLI = 0.95; RMSEA = 0.05 (90% CI: 0.04, 0.05); SRMR = 0.06. Factor loadings of the latent constructs onto individual items are detailed in Table 2.

**TABLE 2:** Confirmatory factor structure of the 7–factor BANPS–GL model.

	Item	SEEK	LUST	PLAY	CARE	FEAR	SADNESS	ANGER
SEEK	SEEK 1	0.47						
	SEEK 2	0.23						
	SEEK 3	0.54						
	SEEK 4	0.76						
	SEEK 5	0.68						
	SEEK 6	0.55						
LUST	LUST 1		0.70					
	LUST 2		0.65					
	LUST 3		0.61					
	LUST 4		0.78					
	LUST 5		0.54					
PLAY	PLAY 1			0.85				
	PLAY 2			0.43				
	PLAY 3			0.62				
	PLAY 4			0.73				
	PLAY 5			0.41				
	PLAY 6			0.48				
CARE	CARE 1				0.59			
	CARE 2				0.77			
	CARE 3				0.69			
	CARE 4				0.72			
FEAR	FEAR 1					0.80		
	FEAR 2					0.82		
	FEAR 3					0.58		
	FEAR 4					0.45		
	FEAR 5					0.71		
SADNESS	SADNESS 1						0.80	
	SADNESS 2						0.82	
	SADNESS 3						0.58	
	SADNESS 4						0.45	
	SADNESS 5						0.71	
	SADNESS 6						0.87	
ANGER	ANGER 1							0.63
	ANGER 2							0.55
	ANGER 3							0.51
	ANGER 4							0.78
	ANGER 5							0.72
	ANGER 6							0.60

*N* = 626; cross–loadings of items were set to 0; all factor loadings *p* < 0.001.

**FIGURE 1:**
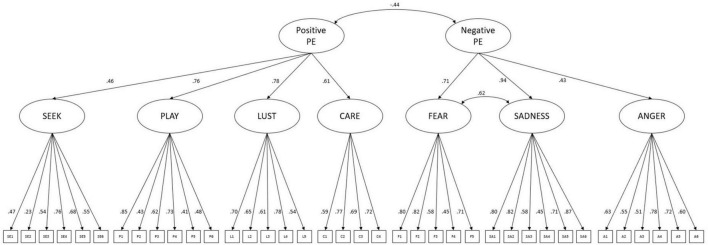
2–higher–order–model of the BANPS–GL. PE, primary emotions.

**FIGURE 2:**
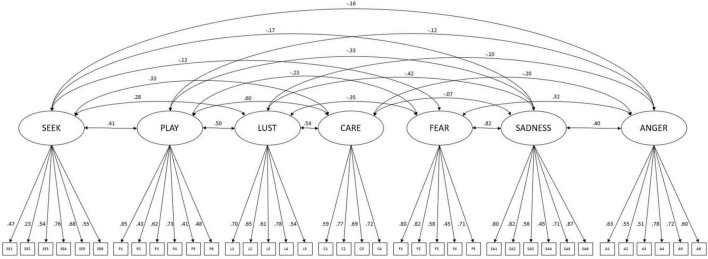
7–factor–model–of the BANPS–GL.

Furthermore, a correlated 2–higher–factor model was examined with the latent construct *positive emotions* predicting positive affects (SEEK, LUST, PLAY, CARE) and *negative emotions* loading onto negative affects (FEAR, SADNESS, ANGER), which showed a slightly worse but still acceptable fit [TLI = 0.93; RMSEA = 0.05 (90% CI: 0.05, 0.05); SRMR = 0.06]. However, this model exhibited a Heywood case due to a negative variance of SADNESS indicating misspecification. The Heywood case was resolved in a third model specifying a correlation between FEAR and SADNESS (*r* = 0.62; *p* < 0.001). The third model showed the same global fit indices as the second model [TLI = 0.93; RMSEA = 0.05 (90% CI: 0.05, 0.06); SRMR = 0.07]. In this model *positive* and *negative emotions* were negatively correlated (*r* = −0.43; *p* < 0.001). All negative affect subscales were significantly predicted by *negative emotions* with loadings ranging from β = 0.43 for ANGER to β = 0.94 for SADNESS (all *p* < 0.001). Factor loadings for *positive emotions* ranged from β = 0.46 (SEEK) to β = 0.79 (LUST; all *p* < 0.001). As highlighted in Figure 1 the pattern of factor loadings for individual subscales onto their respective items remained very similar to the results of the 7–factor model (Figure 2).

### Reliabilities and correlations

The overall scale of BANPS showed acceptable to excellent internal consistency ranging from McDonalds ω = 0.70 (CARE) to ω = 0.86 (SADNESS). The new subscale LUST (L–5) exhibited convincing reliability (ω = 0.79). Table 3 illustrates the correlations of the subscales with the overall scale and the intercorrelations between the subscales. The subscales of BANPS showed medium to high positive correlations (Cohen, 1992) with the related subscales of BFI (*r* = 0.22–0.7; *p* < 0.001). FEAR and SADNESS correlated with Neuroticism (*r* = 0.67–0.7; *p* < 0.001), whilst SEEK showed the highest correlation with Openness (*r* = 0.55; *p* < 0.001). Extraversion indicated a high correlation with PLAY (*r* = 0.53; *p* < 0.001). Likewise, LUST demonstrated the highest association with this scale (*r* = 0.43; *p* < 0.001). Strong correlations were observed for LUST scale and SSSS (*r* = 0.53; *p* < 0.001). Regarding the examination of the distribution characteristics which was based on the Kolmogorov–Smirnov adaptation test for normal distribution and the analysis of the skewness and kurtosis, it can be assumed that none of the scales exhibited a normal distribution.

**TABLE 3:** Correlations between BANPS-GL and validation instruments BFI and SSSS.

Measurement	Variable	LUST	SEEK	CARE	PLAY	FEAR	ANGER	SADNESS	Positive PE	Negative PE
LUST	LUST	–								
BANPS	SEEK	0.19**	–							
	CARE	0.40**	0.22**	–						
	PLAY	0.38**	0.29**	0.44**	–					
	FEAR	−0.28**	−0.06	0.02	−0.15**	–				
	ANGER	−0.78*	−0.11**	−0.15**	−0.08*	0.26**	–			
	SADNESS	−0.35**	−0.10**	−0.06	−0.24**	0.68**	0.34**	–		
	Positive PE	0.74**	0.56**	0.75**	0.74**	−0.18**	−0.15**	−0.27**	–	
	Negative PE	−0.30**	−0.12**	−0.08*	−0.20**	0.83**	0.66**	0.86**	−0.25**	–
BFI	O	0.15**	0.55**	0.22**	0.28**	−0.00	−0.05	−0.04	0.40**	−0.04
	C	0.15**	0.29**	0.22**	0.04	−0.19**	−0.27**	−0.28**	0.24**	−0.31**
	E	0.43**	0.27**	0.40**	0.53**	−0.32**	−0.04	−0.38**	0.58**	−0.32**
	A	0.26**	0.21**	0.49**	0.36**	−0.12**	−0.50**	−0.22**	0.47**	−0.35**
	N	−0.36**	−0.19**	−0.09*	−0.25**	0.70**	0.46**	0.67**	−0.32**	0.78**
SSSS	SSSS	0.53**	0.08*	0.19**	0.27**	−0.17**	0.14**	−0.07	0.40**	−0.04
Age		−0.07	0.05	−0.09*	−0.14*	−0.23**	0.02	−0.17**	−0.08	−0.17**
	M	3.75	3.87	3.78	3.66	3.41	2.55	2.91	3.76	2.22
	SD	0.80	0.60	0.76	0.68	0.87	0.82	0.87	0.50	0.51
	McDonald’s ω	0.80	0.72	0.70	0.76	0.84	0.80	0.86	0.67	0.75
	Skew	−0.64	−0.18	−0.48	−0.38	−0.31	0.33	0.50	−0.42	−0.10
	Kurtosis	0.23	−0.42	−0.36	−0.13	−0.34	−0.29	−0.44	0.20	−0.02
	p^+^	0.00	0.00	0.00	0.00	0.00	0.00	0.00	0.17	0.41

*N* = 926. **p < 0.001, *p < 0.01; Positive PE, positive primary emotions; Negative PE, negative primary emotions; BFI, big five inventory; O, Openness; C, Conscientiousness; E, Extraversion; A, Agreeableness; N, Neuroticism; SSSS, Sexual Sensation Seeking Scale; ^+^Kolmogorov–Smirnov Test.

Finally, both total scales (positive and negative primary emotions) were correlated with extroversion and neuroticism. Results indicate a strong relationship between negative primary emotions and neuroticism (*r* = 0.78) and a moderate negative relationship with extroversion (*r* = −0.32). In contrast, positive primary emotions showed a moderate negative correlation with neuroticism (*r* = −0.32) and a strong positive correlation with extraversion (*r* = 0.58; all *p* < 0.001).

### Gender differences

Gender differences were assessed via multivariate analysis of variance. Detailed results are shown in Table 4. No significant gender differences were observed regarding PLAY, SEEK and ANGER (all *p* > 0.01). Significant differences (all *p* < 0.001) between diverse, female and male participants were found in SADNESS (*F*_2_,_923_ = 22.82; ηp^2^ = 0.05; diverse > female > male), CARE (*F*_2_,_923_ = 8.60; ηp^2^ = 0.02; female > male = diverse), FEAR (*F*_2_,_923_ = 46.69; ηp^2^ = 0.09; diverse > female > male), LUST (*F*_2_,_923_ = 30.12; ηp^2^ = 0.06; male > female > diverse), positive primary emotions (*F*_2_,_923_ = 7.20; ηp^2^ = 0.02; female = male > diverse) and negative primary emotions (*F*_2_,_923_ = 25.40; ηp^2^ = 0.05; diverse = female > male).

**TABLE 4:** Gender differences (MANOVA) in BANPS-GL.

Measures	Female (*n* = 648)		Male (*N* = 257)		Diverse (*n* = 21)		F_2.923_	η p^2^
	***M***	**SD**	***M***	**SD**	***M***	**SD**		
PLAY	3.64	0.67	3.72	0.69	3.37	0.58	3.08	0.01
SEEK	3.87	0.60	3.88	0.60	3.89	0.67	0.03	0.00
ANGER	2.56	0.80	2.53	0.87	2.53	0.84	0.11	0.00
SADNESS	3.00	0.84	2.63	0.88	3.52	0.86	22.82**	0.05
CARE	3.84	0.74	3.66	0.74	3.36	0.97	8.60**	0.02
FEAR	3.55	0.81	2.99	0.88	4.00	0.86	46.69**	0.09
LUST	3.66	0.78	4.03	0.75	3.02	1.01	30.12**	0.06
Positive PE	3.75	0.49	3.82	0.52	3.41	0.54	7.20**	0.02
Negative PE	2.28	0.47	2.04	0.55	2.51	0.52	25.40**	0.05

*N* = 926. **p < 0.001 *p < 0.01; Positive PE, positive primary emotions; Negative PE, negative primary emotions.

### Associations between BANPS-GL and BFI

To further investigate the independent associations between the Big Five traits and primary emotions Table 5 details seven hierarchical multiple regressions controlled for age and gender effects with primary emotions as criteria and BFI as well as SSSS scales as predictors.

**TABLE 5:** Associations between BANPS-GL, SSSS, and BFI controlled for age and gender.

Variable	PLAY			SEEK			LUST			CARE			FEAR			ANGER			SADNESS		
	**R^2^**	**ΔR^2^**	**β**	**R^2^**	**ΔR^2^**	**β**	**R^2^**	**ΔR^2^**	**β**	**R^2^**	**ΔR^2^**	**β**	**R^2^**	**ΔR^2^**	**β**	**R^2^**	**ΔR^2^**	**β**	**R^2^**	**ΔR^2^**	**β**
	**0.02**			**0.06**			**0.06**			**0.02**			**0.13**						**0.05**		
Age			**−0.14**									−0.08			−0**.19**						
Female												**0.10**			**0.26**						**0.19**
Male									**0.21**												
Diverse									−0**.12**			−0.06			**0.08**						**0.09**
**Step 2**	**0.34**	**0.32**		**0.35**			**0.41**	**0.36**		**0.38**	**0.36**		**0.53**	**0.41**		**0.62**			**0.48**	**0.43**	
Age			**−0.16**									−0.05			**−0.12**						
Female												0.06			0.06						0.05
Male									0.04												
Diverse									−0.03			−0.04			0.01						0.02
O			**0.16**			**0.49**			0.02			0.05			0.06						
C			−0**.11**			**0.17**			0.05			**0.09**			0.02			−0.01			−0.05
E			**0.46**			**0.09**			**0.18**			**0.29**			−0**.07**						**−0.16**
A						0.01			**0.14**			**0.44**			**0.12**			−0**.38**			0.03
N			**−0.08**			−0.03			**−0.14**			**0.22**			**0.68**			**0.35**			**0.60**
SSSS			**0.09**			0.03			**0.44**			**0.15**			−0.03			**0.19**			**0.08**

*N* = 926. In bold: <0.01; female was dummy coded as: female = 1; male and diverse = 0; male was dummy coded as male = 1; diverse and female = 0; diverse was dummy coded as: diverse = 1; male and female = 0; BFI, big five inventory; O, Openness; C, Conscientiousness; E, Extraversion; A, Agreeableness; N, Neuroticism; SSSS, Sexual Sensation Seeking Scale.

It was possible to explain 34% of the variance of PLAY via age (β = −0.16), openness (β = 0.16), consciousness (β = −0.11), extraversion (β = 0.46), neuroticism (β = −0.08) and SSS (β = 0.09). 35% of the variance of SEEK was explained by openness (β = 0.49), consciousness (β = 0.17) and extraversion (β = 0.09). LUST exhibited associations with extraversion (β = 0.18), agreeableness (β = 0.14), neuroticism (β = −0.14), and SSS (β = 0.44). These variables collectively explained 41% of the overall variances observed. CARE was linked to extraversion (β = −0.07), agreeableness (β = 0.44), neuroticism (β = 0.22) and SSS (β = 0.15) which in sum explained 38% of the variance. In terms of FEAR, the results indicated significant associations with age (β = −0.12), extraversion (β = −0.07), agreeableness (β = 0.12), and neuroticism (β = 0.68) with 53% explained variance. Furthermore, ANGER demonstrated significant relationships (*R*^2^ = 0.62) with agreeableness (β = −0.38), neuroticism (β = 0.35), and SSS (β = 0.19). Finally, SADNESS was found to be significantly associated with extraversion (β = −0.16), neuroticism (β = 0.60), and SSS (β = 0.08), collectively explaining 48% of the variance of this affective trait.

### Invariance analysis

Lastly, we conducted an invariance analysis of the BANPS-GL regarding across gender. However, due to the relatively small size of the *diverse* sample (*n* = 21), we had to exclude these participants from this specific analysis resulting in total sample of 905 participants. As displayed in Table 6 the 7-factor solution of the BANPS-GL exhibited scalar invariance for both male and female participants.

**TABLE 6:** Fit statistics and comparison of the correlated 7-factor solution of the BANPS-GL across gender.

Model	χ^2^	df	Model comparison	CFI	Δ CFI*	Decision
M1: Configural Invariance	2179.92	1,288		0.961		
M2: Metric Invariance	2291.90	1,319	M1	0.958	−0.003	Accepted
M3: Scalar Invariance	2537.40	1,350	M2	0.948	−0.01	Accepted

CFI, comparative fit index; *ΔCFI ≤ −0.01 signals lack of invariance targeted by the respective comparison of nested models.

## Discussion

The aim of the present work was to translate and validate a self–report measurement for all seven primary emotions into German language, as to date no standardized questionnaires for the German speaking area exists which operationalizes all seven primary emotions. Based on our results, it was confirmed that the German version of the BANPS with its inclusion of an additional LUST scale (BANPS–GL) demonstrated overall convincing psychometric properties. Furthermore, it is possible to measure all seven primary emotion dispositions as originally conceptualized by Panksepp (1998) in a concise and economically valid manner.

The values of the internal consistency, which were in an acceptable to good range for the final version with 38 items, indicate a satisfactory level of reliability (Rost, 2004; Bühner, 2011). The factorial structure obtained through exploratory factor analysis with 38 items resulting in seven factors could be confirmed, under consideration of the restrictions of the factor SADNESS, in a subsequent confirmatory factor analysis. Thereby, the confirmatory factor analysis resulted in a 7–factor model as well as a 2–higher–factor model which both showed good model fit. Compared to Barrett et al. (2013) both models achieved considerably better fit indices. The deviations regarding model fit compared to the original BANPS study could be traced back to the different estimation methods used for the respective CFAs. While Barrett et al. (2013) employed Maximum Likelihood (ML), the present study used the Diagonal Weighted Least Squares (DWLS). Typically, ML underestimates the model fit for ordinally scaled indicators and affects the accuracy of the estimation method for discontinuous and not uniformly distributed data (Mîndrilă, 2010). In contrast, DWLS showed in comparative studies that it provides more accurate factor loading estimates for ordinally scaled data under various conditions (Li, 2016).

Even though Panksepp postulated different neural and behavioral systems for each dimension, representing the different functions as well as anatomy, the individual systems could confluence with each other. This results in an interactive process to enhance the adaptability of individual feelings, perceptions, thoughts and behaviors (Panksepp, 2005, 1998). Therefore, it is not surprising that the independent scales indicated some intercorrelations.

One ambiguity in the individual factors that Barrett et al. (2013) already struggled with in his developmental studies is SADNESS. As repeatedly demonstrated, this system loads on both a stand–alone factor and the factor FEAR, making it impossible to clearly delineate this component statistically (Davis et al., 2003; Barrett et al., 2013; Pascazio et al., 2015; Giacolini et al., 2017; Montag et al., 2019). Due to the close association of SADNESS and FEAR to Neuroticism of the Big Five, the striking correlation is not surprising. Nevertheless, a distinction can be drawn between the two constructs on a psychological and neurological level. Even though the brain structures are close to each other, they are anatomically distinguishable. Likewise, the systems are controlled relatively distinctive at the neurochemical level (Panksepp and Biven, 2012). Due to these factors, the respective subscales of the BANPS–GL were preserved as independent components. Nevertheless, further research should focus on a possible better–defined distinction as well as a renewed detailed examination of the individual items in order to possibly represent SADNESS and FEAR as two independent factors in the future (Pedersen et al., 2014).

The Big Five is one of the most established models to conceptualize different factors of personality (John et al., 1991; Montag and Panksepp, 2017). With regard to the robust correlations between primary emotions and the Big Five, the present results are in line with previous studies and meta–analyses (Davis et al., 2003; Davis and Panksepp, 2011; Barrett et al., 2013, 2010; Marengo et al., 2021). It was demonstrated that Openness for Experience is strongly related to SEEKING, while Extraversion showed high expressions of PLAY. In turn, high levels of Agreeableness were associated with low levels in the ANGER system, confirming the stability of correlation patterns of both the ANPS and BANPS in relation to the Big Five. Largely in line with Barrett et al. (2013), both higher order factors (positive and negative primary emotions) showed the expected relationships with extraversion and neuroticism, underscoring the high conceptual overlap between negative primary emotions and neuroticism as well as positive primary emotions and extraversion. In comparison to the initial findings of Barrett et al. (2013), the integration of LUST into the positive primary emotions factor seems to have further increase its relationship with extraversion (*r* = 0.47 vs. *r* = 0.58) while the relationship between neuroticism and negative primary emotions remained steady (Barrett et al., 2013: *r* = 0.74 vs. *r* = 0.78). To investigate further aspects of convergent validity future studies should examine both higher order factors in relation to the *Positive Affect Negative Affect Scale* (PANAS; Watson et al., 1988).

Regarding the survey of the emotion LUST, it is interesting to consider how the items of SSSS and the L-scale differ. As explained in the Psychometric Assessment, the items of the SSSS are based on the quest of new experience and risky behavior (Kalichman, 1994). In contrast, the L–scale was designed by Fuchshuber et al. (2022) along the lines of Panksepp’s conceptualization of primary affect LUST. Hence, its items assess the dispositions toward feelings of eroticism, sexual pleasure and enjoyment (Fuchshuber et al., 2022). With respect to the diversity yet contentwise connectedness of both SSS and LUST the assessed moderate to strong correlation appears plausible and underscores the criterion validity of the L-scale. In correspondence to this, the present data suggests that the L-scales scales are distinguished by high reliability, satisfying structural validity and plausible correlations with external criteria. Therefore, this study might serve as vital groundwork for a standardized operationalization of LUST. However, more research will be necessary to further evaluate this instrument, especially regarding its external validity and its applicability in clinical populations.

With respect to gender differences, the current study yielded findings that parallel those of Barrett et al. (2013) and Fuchshuber et al. (2022), demonstrating that females exhibited higher scores in CARE, SADNESS, and FEAR, while displaying lower scores in LUST, with effect sizes ranging from small to moderate. Interestingly, no difference was observed between genders in terms of ANGER, which has frequently been documented as higher in males compared to females (Barrett et al., 2013; Montag et al., 2016). Generally, this study indicates decreased positive emotions in diverse and higher negative emotions in diverse and females. Until now, little research has been done with regard to the affective profile of non-binary or trans participants. Therefore, future studies should delve more deeply in this rather inhomogeneous group.

In terms of measurement invariance, the results imply that the BANPS-GL is functioning equivalently across both female and male participants allowing for meaningful comparisons and conclusions.

### Limitations and future perspectives

The present study is limited by components of its sample. Although an attempt was made to ensure diversity, the survey via online questionnaire predominantly addressed the target group of young adults. Thereby, the sample consisted mainly of healthy students from Austrian and German universities. In this respect, it would be useful to evaluate the BANPS–GL in clinical populations as well, to be able to make a more significant statement about the etiological relevance. Since the study placed a great emphasis on the sexual components, a more diverse population of various gender identities as well as sexual orientations would be of importance. Differentiation was collected in the study, but there is no balanced distribution in terms of gender and sexual orientation. Similarly, the generalizability of the results are limited by the large proportion of highly educated females which were investigated. In order to generate norm data for the BANPS-GL future studies will aim to assess larger and more representative samples with education, occupation and gender distributions which show a closer correspondence to the general population.

## Conclusion

This study aimed to develop a questionnaire able to capture primary emotions in their entirety. The presented data as well as consistent previous research findings indicate that the BANPS–GL is characterized by satisfactory structural validity as well as high reliability. With the BANPS–GL an adequate instrument can now be utilized for purposes as delving deeper into the research of the interrelationships between primary emotions and other psychological constructs as well as psychiatric disorders. While the limitations from this sampling discussed above imply the need for further research regarding standardization and broader sampling, the results of this first examination indicate that the BANPS–GL is a reliable, valid and, above all, an economical self–assessment instrument, suitable for quantitative-empirical research.

## Ethics statement

The studies involving human participants were reviewed and approved by the University of Graz. The patients/participants provided their written informed consent to participate in this study.

## Author contributions

JF and H-FU conceptualized the study. TP, DA, LR, and BS collected the data. TP and JF conducted all statistical analyses. TP wrote the first draft of the manuscript. JF, DA, LR, BS, AF, AS, and H-FU read the manuscript and made some critical comments. All authors contributed to the article and approved the submitted version.
