# Supplementary material for: The German version of the brief affective neuroscience personality scales including a LUST scale (BANPS–GL)
Source: Front Hum Neurosci. 2023 Jul 7;17:1213156. doi: 10.3389/fnhum.2023.1213156 (PMC10359993; doi:10.3389/fnhum.2023.1213156)
Supplement: Supplementary file 1 [file Table_1.docx]

**The German Version of the Brief Affective Neuroscience Personality Scales including a LUST Scale (BANPS–GL)**

**SUPPLEMENTARY MATERIAL**

S1

Instruction and Items for the Brief Affective Neuroscience Personality Scales including a LUST Scale (BANPS-GL) 38 item version in German language together with a short manual.

**Instruction**

Geben Sie auf der nachstehenden Skala an, wie sehr Sie den einzelnen Aussagen zustimmen oder nicht zustimmen. Bitte lesen Sie jede Aussage sorgfältig durch und antworten Sie so ehrlich wie möglich.

**List of Items**

| Nr. | Dimension | Item |
| --- | --- | --- |
| 1 | P | Leute, die mich kennen, würden sagen, dass ich ein sehr lebenslustiger Mensch bin. |
| 10 | P | Ich bin sehr verspielt. |
| 15 | P | Ich albere gerne mit anderen Leuten herum. |
| 22 | P | Ich bin jemand, der leicht zu amüsieren ist und viel lacht. |
| 25 | P | Ich mag es nicht besonders, Witze zu machen und „Witzeleien“ auszutauschen.* |
| 33 | P | Meine Freunde würden mich wahrscheinlich als zu ernsthaft beschreiben.* |
| 2 | A | Wenn ich frustriert bin, werde ich normalerweise wütend. |
| 5 | A | Ich werde selten so wütend, dass ich jemanden schlagen möchte.* |
| 12 | A | Meine Freunde würden mich wahrscheinlich als Hitzkopf beschreiben. |
| 23 | A | Menschen, die mich gut kennen, würden sagen, dass ich fast nie wütend werde.* |
| 27 | A | Ich werde selten so wütend auf jemanden, dass ich ihn anschreien möchte.* |
| 30 | A | Wenn mich jemand wütend macht, neige ich dazu, lange Zeit aufgebracht zu bleiben. |
| 3 | S | Ich bin nicht sehr neugierig.* |
| 24 | S | Ich bin normalerweise nicht daran interessiert, Probleme und Rätsel nur um des Lösens willen zu lösen.* |
| 29 | S | Ich bin kein besonders wissbegieriger Mensch.* |
| 32 | S | Meine Neugier treibt mich an, Dinge zu tun. |
| 36 | S | Ich genieße es, neue Lösungen für Probleme zu finden. |
| 38 | S | Ich denke gerne über den Tellerrand hinaus. |
| 4 | C | Ich bin ein Mensch, der andere gern berührt und umarmt. |
| 16 | C | Ich habe oft das Bedürfnis, mich um Menschen zu kümmern, die mir am nächsten stehen. |
| 18 | C | Ich bin nicht besonders zärtlich.* |
| 31 | C | Ich möchte nicht unbedingt, dass mir jemand emotional nahe steht.* |
| 6 | F | Ich mache mir selten Sorgen über meine Zukunft.* |
| 17 | F | Ich mache mir oft Sorgen über die Zukunft. |
| 19 | F | Es gibt sehr wenige Dinge, die mich beunruhigen.* |
| 26 | F | Ich kann manchmal nicht aufhören, mir über meine Probleme Gedanken zu machen. |
| 34 | F | Ich habe sehr wenige Ängste in meinem Leben.* |
| 8 | SA | Ich werde selten betrübt.* |
| 9 | SA | Ich erlebe selten Traurigkeit oder Verzweiflung.* |
| 11 | SA | Ich habe oft das Gefühl, dass ich gleich weinen werde. |
| 13 | SA | Ich fühle mich nicht sehr oft einsam.* |
| 20 | SA | Ich fühle mich oft einsam. |
| 37 | SA | Ich fühle mich oft traurig. |
| 7 | L | Mir fällt es leicht, mich erotischen Erfahrungen hinzugeben. |
| 14 | L | Mit meiner Sexualität habe ich häufig schlechte Erfahrungen gemacht.* |
| 21 | L | Ich empfinde meine Sexualität allgemein als befriedigend. |
| 28 | L | Ich stehe Sexualität nicht besonders offen gegenüber.* |
| 35 | L | Ich kann das Ausüben von sexuellen Handlungen (Geschlechtsverkehr, Masturbation, etc.) voll und ganz genießen. |

*Note*. Nr. = Item number in the Questionnaire; P = PLAY, A = ANGER, S = SEEKING, C = CARE, F = FEAR, SA = SADNESS, L = LUST; Likert scale: 1: Starke Ablehnung – 5: Starke Zustimmung. Items marked with * are reverse coded.

**Evaluation scheme**

| Dimension | Item number |
| --- | --- |
| PLAY (P) | 1, 10, 15, 22, 25*, 33* |
| ANGER (A) | 2, 5*, 12, 23*, 27*, 30 |
| SEEK (S) | 3*, 24*, 29*, 32, 36, 38 |
| CARE (C) | 4, 16, 18*, 31* |
| FEAR (F) | 6*, 17, 19*, 26, 34* |
| SADNESS (SA) | 8*, 9*, 11, 13*, 20, 37 |
| LUST (L) | 7, 14*, 21, 28*, 35 |

*Note.* * = reverse coded.

**Likert Scale**

1 = Starke Ablehnung

2 = Ablehnung

3 = Weder Ablehnung noch Zustimmung

4 = Zustimmung

5 = Starke Zustimmung

S2

Please retrieve the Instruction and Items for the Brief Affective Neuroscience Personality Scales (BANPS) 33 item version in English language together with a manual from Appendix of the BANPS:

Barrett, F. S., Robins, R. W., & Janata, P. (2013). A brief form of the Affective Neuroscience Personality Scales. *Psychological Assessment*, *25*(3), 843. https://doi.org/10.1037/a0032576

Instruction and Items for the L-Scale 5 Item Version in English language together with a short manual. As in the German version of the BANPS-GL, we recommend that the LUST dimension be incorporated in steps of 7. The order of the BANPS subscales thus remains identical.

**Instruction**

Use the scale shown below to indicate how much you agree or disagree with each statement. Please read each statement carefully and respond as honestly as possible.

**List of Items**

| Nr. | Dimension | Item |
| --- | --- | --- |
| 7 | L | For me, it is easy to indulge myself in erotic experiences. |
| 14 | L | I have often had negative experiences with my sexuality.* |
| 21 | L | I am generally satisfied with my sexuality. |
| 28 | L | I am not particularly open about sexuality.* |
| 35 | L | I can fully enjoy engaging in sexual activities (intercourse, masturbation, etc.). |

*Note*. Nr. = Item number in the Questionnaire; L = LUST; Likert scale: 1: Strongly disagree – 5: Strongly agree. Items marked with * are reverse coded.
